# The effect of iontophoresis delivery of fluoride in stannous fluoride desensitizing toothpaste on dentin permeability in human extracted teeth

**DOI:** 10.1038/s41598-022-18043-9

**Published:** 2022-08-10

**Authors:** Kanittha Kijsamanmith, Nutchaya Monthonjulaket, Nalina Kuanpradit, Kanlisa Thongwong, Nattawat Kijprasert

**Affiliations:** Department of Oral Biology, Faculty of Dentistry, Mahidol University, Yothi Road, Bangkok, 10400 Thailand

**Keywords:** Health care, Medical research

## Abstract

This study aimed to determine the effect of iontophoresis delivery of fluoride in stannous fluoride (SnF_2_) toothpaste on dentin permeability in human extracted third molars. For dentin permeability test, 26 dentin specimens were randomly divided into 4 groups; SnF_2_ without-iontophoresis (*n* = 10), SnF_2_ with-iontophoresis (*n* = 10), no SnF_2_ without-iontophoresis (*n* = 3), and no SnF_2_ with-iontophoresis (*n* = 3). The hydraulic conductance of dentin (HD) was measured after smear layer removal, immediate treatment, 7 days, and acid challenge. The other 26 specimens were also prepared under these different conditions to assess degree of dentinal tubule occlusions using scanning electron microscopy (SEM). Percentage decrease of HD in SnF_2_ without-iontophoresis after immediate treatment, 7 days and acid challenge were 38.38 ± 13.61, 56.92 ± 17.22 and 33.07 ± 23.57%. The corresponding values in SnF_2_ with-iontophoresis were 42.16 ± 14.49, 62.35 ± 15.67 and 50.01 ± 12.60%, respectively. There was a significant difference between without- and with-iontophoresis groups after acid challenge (*p* < 0.05). For SEM, after 7 days, SnF_2_ with-iontophoresis showed deeper dentinal tubule occlusion (*p* < 0.05) and more resistance to acid challenge than SnF_2_ without-iontophoresis. No significant change was observed in groups of no SnF_2_ treatment. Cathode iontophoresis could enhance the effect of SnF_2_ toothpaste in decreasing dentin permeability and increasing resistance to acid challenge.

## Introduction

Dentin hypersensitivity is a painful dental problem which is characterized by short sharp pain arising from exposed dentin in response to stimuli such as evaporative, chemical, osmotic, thermal and tactile, leading to a negative impact on the patient’s quality of life^1,2^. The most accepted mechanism of dentin hypersensitivity are based on the hydrodynamic theory which proposed by Branström^3^. Hydrodynamic stimuli which are applied to dentin could produce an outward or inward movement of dentinal fluid flow through dentin^4,5^, and lead to exciting the intradental nerve in the dentin and/or in the dental pulp to elicit dental pain^6,7^. Thus, based on the hydrodynamic theory, degree of dentin sensitivity depends upon amount of exposed dentinal tubules and dentinal fluid flow through dentin or dentin permeability which are measured as hydraulic conductance^8,9^. Many acidic foods such as fruits containing citric acid, tartaric acid, mangiferonic acid, which have strong erosive ability to remove smear layer from exposed dentin, could significantly increase hydraulic conductance of dentin and probably lead to dentin hypersensitivity^10,11^.

At present, two main management strategies of dentin hypersensitivity consists of nerve desensitization and dentinal tubule occlusion^12^. Many desensitizing agents such as strontium acetate, potassium oxalate, milk and CPP-ACP have a good ability to occlude the dentinal tubules and reduce the hydraulic conductance of dentin; hence, dentinal tubule occlusion is an effective method to treat dentin hypersensitivity^13,14^. Recently, Hines et al.^15^ demonstrated that stannous fluoride toothpaste is another desensitizing agent which provides a significant reduction in dentin hypersensitivity due to its ability to occlude dentinal tubules by formation of an insoluble metal compound that precipitates in dentinal tubules. Additionally, SnF_2_ toothpaste showed a greater anti-erosive potential and provided a significant effect on the reduction of dentin hypersensitivity when compared to the control toothpastes^16^.

According to consensus-based recommendations of the Canadian advisory board on dentin hypersensitivity in 2003, desensitizing fluoride toothpaste should be considered as a non-invasive first line of at-home treatment for reducing dentin hypersensitivity^17^. Furthermore, iontophoresis of 2% sodium fluoride solution can be used as in-office desensitizing treatment due to its immediate and long-lasting desensitizing effect when compared to HEMA-G and topical fluoride application^18–20^. In cathode fluoride-iontophoresis, fluoride ions are repelled by a cathode electric current, and attracted to a positively charged tooth which is connected to an anode electrode^21^. Huang and Guo^22^ found that 3 times application of 5 min 2% sodium fluoride-iontophoresis showed larger particle size and deeper penetration of fluoride particles in dentinal tubules, when compared to fluoride treatment without iontophoresis.

However, there were no iontophoresis studies which focus on the effect of iontophoresis transport of fluorides from SnF_2_ toothpaste across human dentin on dentinal fluid flow and acid resistance. Frequency and duration of treatment would be an important factor for a greater reduction in dentin permeability as a longer repeated treatment period might increase tubule occlusion of desensitizing agent^14,22^. Furthermore, an ideal desensitizing agent should against the acidic diets to maintain its occluding ability in dentinal tubules^14^. Therefore, the objective of this study was to determine the effect of iontophoresis delivery of fluoride in SnF_2_-desensitizing toothpaste on dentin permeability and tubule occlusion after immediate treatment, after 7-day treatment period and after acid challenge in human extracted teeth using dentin permeability test and scanning electron microscopy (SEM).

## Results

### Percentage changes of hydraulic conductance

**Figure 1:**
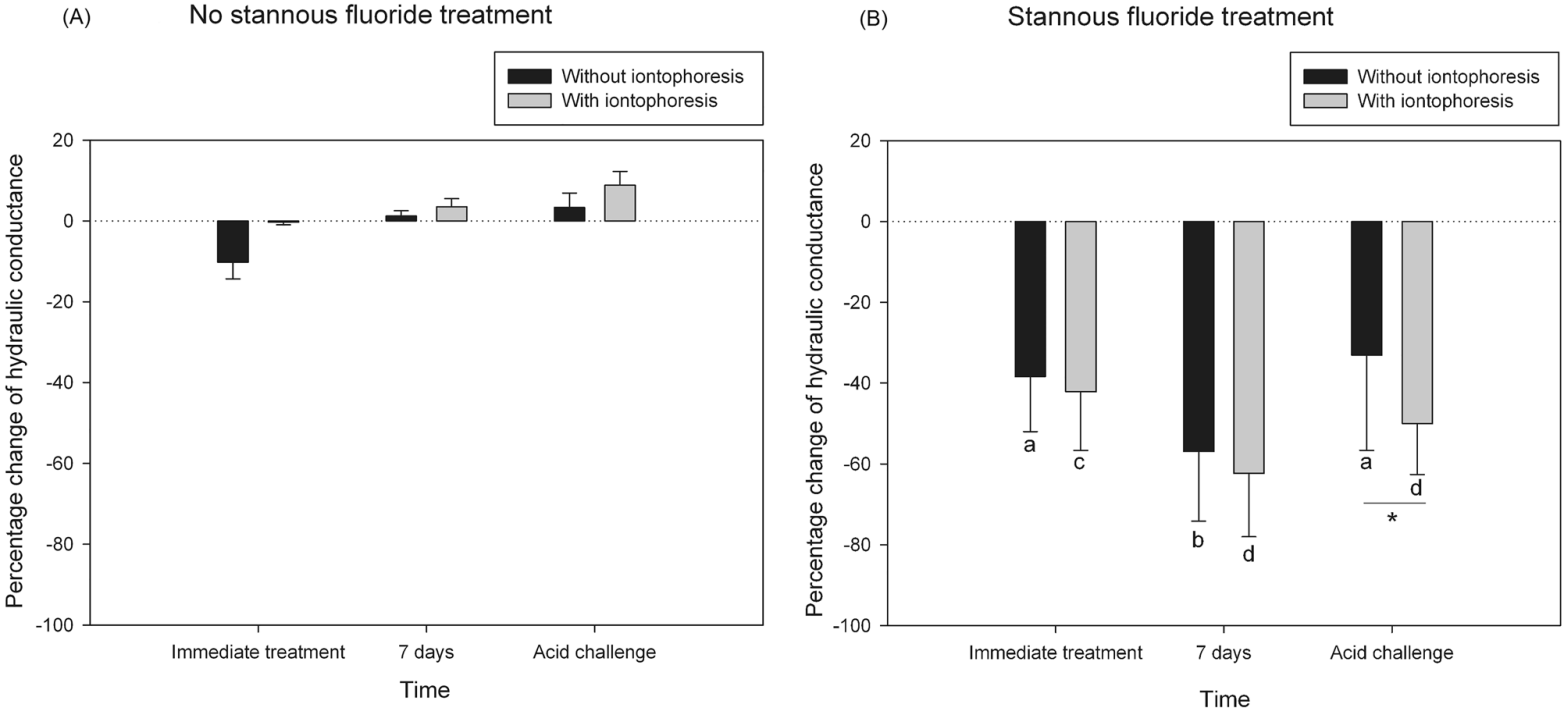
Mean (± 1 SD) percentage decreases of hydraulic conductance of dentin after (**A**) no stannous fluoride treatment; (**B**) stannous fluoride treatment, without (black column) and with (gray column) iontophoresis at immediate treatment, 7 days and acid challenge. The same lowercase letter represents no significant change among the times of treatment. *Statistically significant difference between without and with iontophoresis (*p* < 0.05, 2-way repeated measures ANOVA and Tukey test).

For no SnF_2_ treatment groups, mean percentage changes of HD in no SnF_2_ without-iontophoresis group after immediate treatment, after 7 days and after acid challenge were − 10.22 ± 4.10, 1.21 ± 1.33 and 3.32 ± 3.60%. The corresponding values in no SnF_2_ with-iontophoresis group were − 0.32 ± 0.63, 3.54 ± 1.98 and 8.85 ± 3.41%, respectively (Fig. 1A). There was no significant difference in percentage change of HD among the periods in both groups of no SnF_2_ treatment (*p* > 0.05).

For SnF_2_ treatment groups, mean percentage changes of HD in SnF_2_ without-iontophoresis group after immediate treatment, after 7 days and after acid challenge were − 38.38 ± 13.61, − 56.92 ± 17.22 and − 33.07 ± 23.57%. The corresponding values in SnF_2_ with-iontophoresis group were − 42.16 ± 14.49, − 62.35 ± 15.67 and − 50.01 ± 12.60%, respectively (Fig. 1B). Comparing percentage decrease of HD between SnF_2_ without- and with-iontophoresis group, there was no significant difference after immediate treatment (*p* = 0.614) and after 7 days (*p* = 0.469); however, a statistically significant difference was found after acid challenge (*p* < 0.05).

Comparing among the periods in both groups of SnF_2_ treatment, it was found that there was a statistically significant difference (*p* < 0.001). In SnF_2_ without-iontophoresis group, percentage decrease of HD after 7 days was significantly greater than percentage decrease of HD after immediate treatment and after acid challenge (*p* < 0.05). However, percentage decrease of HD after acid challenge was not significantly different from that after immediate treatment (*p* = 0.603).

In SnF_2_ with-iontophoresis group, percentage decrease of HD after 7 days was significantly greater than that after immediate treatment (*p* < 0.05). Although percentage decrease of HD after acid challenge was lower than that after 7 days but it was not significantly different (*p* = 0.077). 

### SEM findings

**Figure 2:**
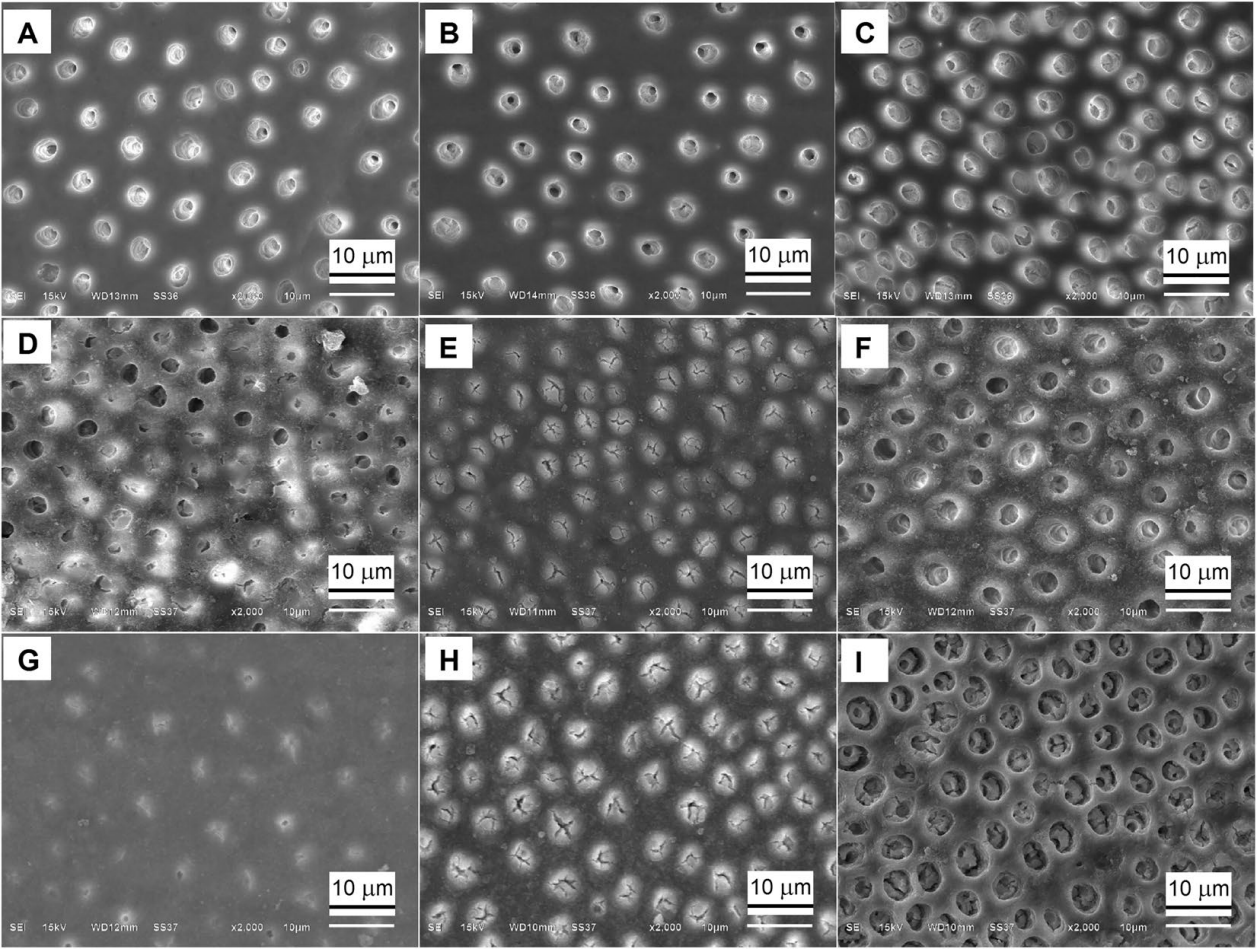
(**A**–**I**) Scanning electron micrographs of cross sectional views of human dentin (original magnification × 2000). (**A**) Treated with 17% EDTA; (**B**) treated with distilled water for 7 days; (**C**) applied with distilled water and cathode iontophoresis for 7 days; (**D**–**F**) applied with stannous fluoride without iontophoresis; (**G**–**I**) applied with stannous fluoride and cathode iontophoresis at (**D**,**G**) immediate treatment, (**E**,**H**) 7 days and (**F**,**I**) acid challenge.

**Figure 3:**
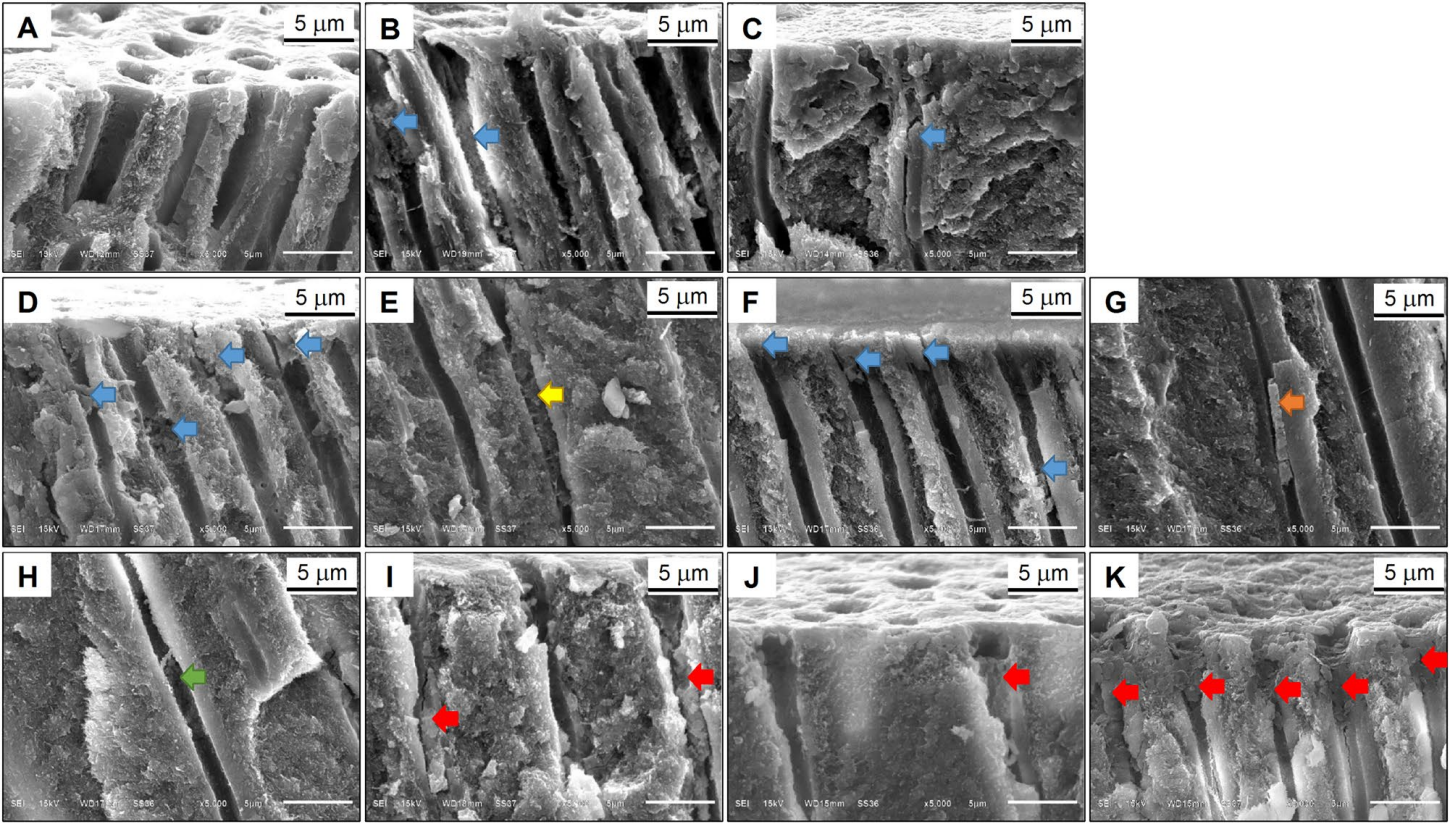
(**A**–**K**) Scanning electron micrographs of longitudinal views of human dentin (original magnification × 5000). (**A**) Treated with 17% EDTA; (**B**,**C**) immediate treatment of stannous fluoride, (**B**) without iontophoresis and (**C**) with iontophoresis, the dentinal tubules were partially blocked with deposits (blue arrows); (**D**–**H**) 7 days of stannous fluoride treatment, (**D**,**E**) without iontophoresis and (**F**–**H**) with iontophoresis, all dentinal tubules were occluded with deposits at dentin surfaces (blue arrow), granular precipitates (yellow arrow) at the depth of 50 µm, bundle precipitates (orange arrow) at the depth of 130 µm, and small granules (green arrow) at the depth of 250 µm from dentin surfaces; (**I**–**K**) after acid challenge of (**I**) without iontophoresis and (**J**,**K**) with fluoride iontophoresis, red arrows showing precipitates still remained within dentinal tubules.

It was observed that dentin without smear layer showed opened tubular orifices and fully patent dentinal tubules (Figs. 2A,3A), and classified as having grade 5 occlusion. In specimens of no SnF_2_ treatment, no tubule occlusion was observed (Fig. 2B,C), and classified as having grade 5 occlusion. In specimens of SnF_2_ without-iontophoresis group, after immediate treatment, fine particles were observed on the dentin surface. Most dentinal tubules were initially occluded (Figs. 2D, 3B), and classified as having grade 3 occlusion. After 7 days of treatment, the tubule orifices were completely blocked with particles (Figs. 2E, 3D), and classified as having grade 1 occlusion. After the acid challenge, 6% citric acid removed some occlusions from the dentinal surface, so the dentinal tubules remained partially occluded (Figs. 2F, 3I), and classified as having grade 3 occlusion.

In specimens of SnF_2_ with-iontophoresis group, after immediate treatment, the dentinal tubules were occluded with particles (Figs. 2G, 3C), and classified as having grade 3 occlusion. After 7 days of treatment, the dentinal tubules were completely blocked with particles (Figs. 2H, 3F), and classified as having grade 1 occlusion. Although an acid challenge with 6% citric acid removed some occlusions from the dentinal surface, most occlusion tag still remained blocked within the dentinal tubules (Figs. 2I, 3J,K), and classified as having grade 2 occlusion.

**Figure 4:**
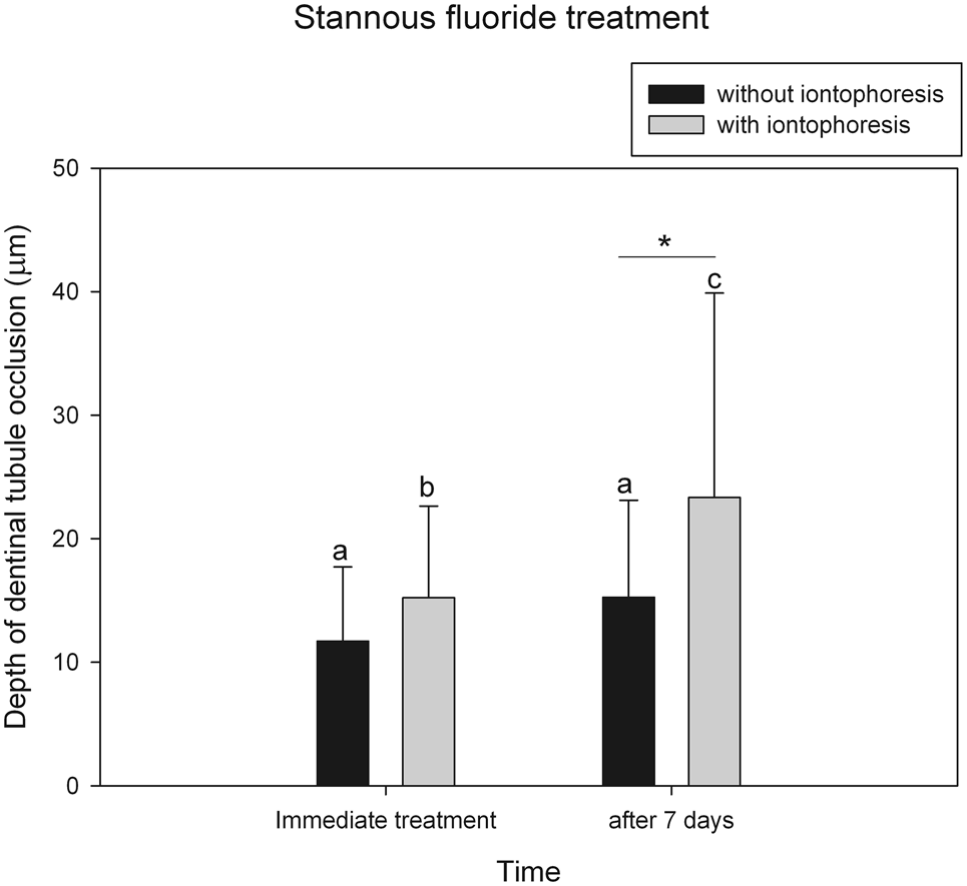
Mean (± 1 SD) depths of dentinal tubule occlusion after stannous fluoride treatment, without (black column) and with (gray column) iontophoresis at immediate treatment and 7 days. The same lowercase letter represents no significant change after 7 days. *Statistically significant difference between groups (*p* < 0.05, 2-way ANOVA and Tukey test).

In both groups of SnF_2_ treatment, the depths of dentinal tubule being occluded after immediate treatment and after 7 days are presented in Fig. 4. After immediate treatment, there was no significant difference in depths of tubule occlusion between SnF_2_ without- and with-iontophoresis groups (*p* = 0.282). However, after 7 days, SnF_2_ with-iontophoresis group showed deeper dentinal tubule occlusion than SnF_2_ without-iontophoresis group (*p* < 0.05). In addition, precipitates in SnF_2_ with-iontophoresis group were greater and penetrated deeper in dentinal tubules than those in SnF_2_ without-iontophoresis group. As shown in SnF_2_ without-iontophoresis specimens (Fig. 3E), particle precipitations with size of 13.46 µm were present in dentinal tubules at a distance of 50 µm from the dentinal surface. Meanwhile, in SnF_2_ with-iontophoresis specimens, the bundle precipitates with size of 8.07 µm blocked in dentinal tubules at the depth of 130 µm (Fig. 3G), and granule precipitates with size of 0.2–0.5 µm were found at depth of 120–250 µm after 7 days of treatment (Fig. 3H).

## Discussion

This study demonstrated that SnF_2_ with-iontophoresis could reduce the HD after acid challenge better than SnF_2_ without-iontophoresis. Consistent with SEM findings, SnF_2_ with-iontophoresis could promote dentinal tubule occlusion deeper and resist to acid challenge better than SnF_2_ without-iontophoresis. The mechanism of action of SnF_2_ treatment on dentin, besides CaF_2_, various mineral salts include Ca(SnF_3_)_2_, Sn_3_F_3_PO_4_, Sn_2_OH(PO_4_), Sn(OH)_2_, and SnH(PO_4_) precipitated and bound on the various dentin structures^23^. Takamizawa et al.^24^ also showed that the dentin specimens treated with 0.4% and 0.454% SnF_2_ toothpastes had greater dentinal tubule occlusion than those treated with distilled water after 4 days of treatment, and there was no significant difference in occlusion between 0.4 and 0.454% concentrations. Based on hydrodynamic theory, the presence of precipitates on the dentin surface and within dentinal tubules could reduce dentinal fluid movement, thereby reducing HD and leading to relieving dentin hypersensitivity^12–14^. In agreement with the results of previous studies^15,16^, our study supported that SnF_2_ treatment both without- and with-iontophoresis could be effective methods for decreasing dentin hypersensitivity.

After SnF_2_ treatment for 7 days, percentage decreases of HD in both groups were significantly greater than those after immediate treatment. Corresponding to the SEM micrographs, the dentin surface became totally occluded (grade 1 occlusion) and showed more precipitates in dentinal tubules in both groups of SnF_2_ treatment. Therefore, increased frequency of treatment caused more surface precipitation and greater tubule occlusion, leading to a greater reduction in HD and a greater benefit for dentin hypersensitivity relief. Randomized clinical studies of Creeth et al.^25^ also indicated that dentin hypersensitivity reduced after single brushing with 0.454% SnF_2_ toothpaste and significantly greater reduced after 3 days twice-daily use. However, after 7 days, SnF_2_ with-iontophoresis group showed deeper dentinal tubule occlusion than SnF_2_ without-iontophoresis group. This implies that SnF_2_ treatment with iontophoresis could help to promote particle precipitation in dentinal tubules better than the treatment without iontophoresis.

Regarding to the effect of fluoride iontophoresis, fluoride ions from stannous fluoride are repelled by a cathode and attract rapidly to a positively charged tooth which is connected to an anode, resulting in increased fluoride penetration at various levels of dentin depth^26^. Considering the safety of the process, applying an electric current 0.5 mA for 5 min was chosen in the study based on previous scientific report that an application of electric current lesser than 1 mA cannot produce histological or ultrastructural changes in the dental pulp^27^. In SEM findings of our study, SnF_2_ with-iontophoresis group achieved more precipitates at greater penetration depth in dentinal tubules than SnF_2_ without-iontophoresis group. Likewise, in previous study of Huang and Guo^22^, 2% sodium fluoride iontophoresis significantly increased the depth of fluoride penetration and the size of granular precipitates, when compared to the treatment without iontophoresis. In addition, McBride et al.^28^ demonstrated that teeth receiving an iontophoresis with 2% sodium fluoride responded with reduced levels of sensitivity when compared to the teeth receiving an iontophoresis with deionized water. Furthermore, many previous studies strongly supported that iontophoresis of 2% sodium fluoride was more effective in reducing dentin hypersensitivity than topical fluoride application alone^21,29^. Hence, these evidences support the use of fluoride iontophoresis to enhance particle precipitation in dentinal tubules and to facilitate a reduction in dentin hypersensitivity.

Recently, Kijsamanmith et al.^30^ demonstrated that fluoride iontophoresis with a neutral 2% sodium fluoride solution had no negative effect on the seal ability of self-etch adhesive in human dentin, when compared to without fluoride iontophoresis. Also, it could improve the seal ability of the self-etch adhesive in intact dentin better than that in caries affected dentin. Thus, the use of 2% sodium fluoride iontophoresis in combination with self-etch adhesive could be an effective in-office technique for reducing dentin permeability and providing a desensitizing effect. Meanwhile, the present study is the first to demonstrate the use of SnF_2_ treatment with iontophoresis for decreasing dentin permeability. Cathode iontophoresis could be an effective alternative method to enhance dentinal tubule occlusion and acid resistance of SnF_2_ toothpaste, therefore improved its desensitizing effect.

Ideally, desensitizing agent should resist the acidic diets or drinks in oral environment for long lasting desensitizing effect. The present study used 6% citric acid solution for the acid challenge^14^, because citric acid is an organic compound and occurs naturally in citrus fruits, including lemons, limes, oranges, grapefruits, strawberries and pineapples. Lemons and limes are the most acidic fruits which have about 5–7% citric acid and their pH between 2.00–2.60. In the study of Kijsamanmith et al.^10^, a single exposure of dentin to tropical fruit juice, especially green mango or lime, for 5 min could increase dentin permeability and have a strong erosive ability to remove the smear layer, similar to an exposure of dentin to 37% phosphoric acid for 30 s. People consuming these tropical fruits and juices could develop dentin hypersensitivity. Otherwise the desensitizing effects may not be permanent, if these acidic fruits and juices dissolve desensitizing products and reopen the dentinal tubules. Thus, the present study used 6% citric acid for 5 min to test the acid resistance of desensitizing agent.

After acid challenge, SnF_2_ without-iontophoresis group showed significant less percentage reduction in HD, when compared to after 7 days. Meanwhile, with-iontophoresis group showed no significant difference in percentage decrease of HD between after acid challenge and after 7 days. Consistent with the SEM micrographs of our study, the precipitates occluding the dentinal tubules of the iontophoresis group were less likely to be affected by the acid challenge than the without-iontophoresis group. In cross-sectional views of dentin specimens, after the acid challenge with 6% citric acid, the dentinal tubules of with-iontophoresis group remained mostly occluded (grade 2 occlusion); whereas, the dentinal tubules of without-iontophoresis group remained partially occluded (grade 3 occlusion). This implies that the treatment with iontophoresis might resist to acid challenge better than the treatment without iontophoresis.

Although all fluoride types are able to help strengthen teeth against high acidity in acidic foods, not all available types of fluoride provide the same level of acid resistance. In situ study of Hooper et al.^31^ supported that stannous fluoride toothpaste could deliver greater protection from erosive acid challenge, when compared with conventional sodium fluoride toothpaste. For anti-erosive property of tin-containing fluoride agents, tin was retained on surface precipitation and within the depth of 10 µm; therefore, it seemed to depend upon the incorporation of tin in the mineralized dentin when the organic portion was preserved^23^. Our study found that, even after an acid challenge, SnF_2_ treatment only could reduce the HD. However, SEM micrographs showed that the dentinal tubules remained partially occluded with deposits (grade 3 occlusion). Meanwhile, SnF_2_ treatment combined with cathode iontophoresis could significantly reduce the HD after acid challenge better than SnF_2_ without-iontophoresis, and revealed that dentinal tubules still mostly occluded (grade 2 occlusion) with precipitates at deeper penetration in dentinal tubules, caused by the faster rate of fluoride diffusion due to repeated fluoride iontophoresis^22,25^.

With limitation in this study, SnF_2_ treatment both without and with fluoride iontophoresis could reduce the HD of human dentin. Additionally, Cathode iontophoresis of SnF_2_ toothpaste could promote dentinal tubule occlusion deeper and resist to acid challenge better than the topical application of SnF_2_ toothpaste alone. Therefore, SnF_2_ treatment with cathode iontophoresis might help to prevent dentin hypersensitivity after taking an acidic food better than SnF_2_ treatment alone.

In conclusion, Cathode iontophoresis could enhance the effect of SnF_2_ toothpaste in decreasing dentin permeability and increasing resistance to an acid challenge.

## Materials and methods

The study was approved by the Institutional Review Board (IRB) of the Faculty of Dentistry and Faculty of Pharmacy at Mahidol University (COE.No.MU-DT-IRB 2020/023.2206), and complied with the principles of the Declaration of Helsinki. The collection of teeth and the experimental methods were performed in accordance with the guidelines and regulations of the IRB, Faculty of Dentistry/Faculty of Pharmacy, Mahidol University. The need for informed consent was waived by the IRB of the Faculty of Dentistry/Faculty of Pharmacy, Mahidol University. Fifty-two intact human third molars without crack or craze line, no history of root canal treatment or crown restoration were obtained from Oral and Maxillofacial clinic, Dental Hospital, Faculty of Dentistry, Mahidol University. The teeth were extracted due to orthodontic purpose, eruption problem, or prophylactic removal. The extracted teeth were immediately kept in 0.1% thymol solution (M Dent, Nakhon Pathom, Thailand, and used in the study within 2 weeks after extraction.

### Specimen preparation

Each tooth was sectioned 3 mm above and below the cemento-enamel junction using a diamond disc with water coolant to achieve the specimen with exposed occlusal dentin surface. After pulpal tissue removal, the pulpal side was etched with 37% phosphoric acid (ScotchBond Etchant, 3 M ESPE; St Paul, MN, USA) for 15 s and rinsed with normal saline solution (NSS) for 30 s. The smear layer covered dentin was removed using 17% EDTA solution (M Dent, Nakhon Pathom, Thailand) for 60 s and rinsed with NSS for 30 s. Thereafter, the crown specimens were randomly divided into two series; series 1: dentin permeability test (*n* = 26) and series 2: SEM study (*n* = 26).

### Dentin permeability test

Following the protocol of Kijsamanmith et al.^14^, each dentin specimen was connected to the fluid filtration system to observe air bubble movement in the capillary of the system with an internal diameter of 300 µm (DADE, Miami, FL, USA) under simulated intrapulpal pressure of 11 mmHg above atmospheric. Distance and time of air bubble movement were calculated for HD as follows: HD = [(distance of air bubble movement × cross sectional area of capillary)/(time of air bubble movement × area of the exposed dentin × pressure]. HD (without smear layer) was measured as the baseline. After that, 26 specimens were randomly divided into 4 groups;

- Group 1: SnF_2_ without-iontophoresis (*n* = 10), 0.429% SnF_2_ toothpaste (1040 ppm; GSK group of companies, UK) was applied on a moist occlusal dentin surface and agitated with an applicator brush for 1 min and left undisturbed for 4 min.
- Group 2: SnF_2_ with-iontophoresis (*n* = 10), occlusal dentin surface was applied with SnF_2_ toothpaste and treated with cathode iontophoresis using an iontophoresis device (DENTAPHOR™-II MODEL 6111D, Life-Tech Inc^®^, USA) at electrical current 0.5 mA for 5 min^30^.
- Group 3: no SnF_2_ without-iontophoresis (*n* = 3), distilled water was applied on a moist occlusal dentin surface and agitated with an applicator brush for 1 min and left undisturbed for 4 min.
- Group 4: no SnF_2_ with-iontophoresis (*n* = 3), occlusal dentin surface was covered with cotton soaked with distilled water and treated with cathode iontophoresis as described in group 2.

After immediate treatment, the dentin surface was cleaned with a moist cotton swab, and HD was measured again. Then, the specimens were immersed in NSS and kept in a humidity chamber (Memmert, Schwabach, Germany) at 37 °C for 7 days. During these 7 days, in group 1, dentin surface was treated with SnF_2_ toothpaste for 5 min daily as described above. Whereas, in group 2, dentin surface was treated with SnF_2_ toothpaste and cathode iontophoresis as mentioned before every other day (3 times of iontophoresis^22^ within 7 days), and alternately treated with SnF_2_ toothpaste without iontophoresis in the other days. In group 3 and 4, dentin surface was applied with distilled water and treated as described in group 1 and 2, respectively. After 7 days, HD was re-measured before and after acid challenge. For acid challenge, the dentin surface was treated with 6% citric acid solution (pH = 2.08) which prepared from citric acid monohydrate (Loba Chemie, Mumbai, India) for 5 min and rinsed with NSS for 30 s. The use of acid challenge with 6% citric acid solution for 5 min represents the acid resistance of desensitizing agent because the desensitizing agent should withstand the acidic diets or drinks in the oral environment^14^.

The percentage (%) change of HD after immediate treatment (imm), after 7 days (7D) and after acid challenge (AC) were calculated by the following formula: The % change of HD (imm/7D/AC) = {[HD (without smear layer) − HD (imm/7D/AC)]/HD (without smear layer)} × 100%

### Scanning electron microscopy

Dentin specimens after smear layer removal and after treatment in each group under different conditions (after immediate treatment, 7 days and acid challenge) were prepared to observe under a scanning electron microscope (JSM-5410 LV; JEOL, Tokyo, Japan) in both cross sectional and longitudinal views. For each specimen, general characteristic of dentin surface, dentinal tubule occlusion and evidence of particle formation in dentinal tubules were observed by 3 independent blinded examiners. The SEM images were assessed for degree of tubule occlusion followed the protocol of Davies et al.^32^ where grades 1, 2, 3, 4 and 5 were defined as 100%, 75%, 50%, 25% and 0% of dentinal tubules being occluded, respectively. The depths of tubule occlusion (µm) were determined from the dentinal surface to the deepest point which the occluding agents could penetrate into each dentinal tubule.

### Statistical analysis

The percentage changes of HD and the depths of tubule occlusion were represented as mean ± standard deviation (SD). Regarding the different treatments and time intervals, the mean percentage changes of HD were compared using two-way repeated measures analysis of variance (ANOVA); meanwhile, the mean depths of tubule occlusion were compared using two-way ANOVA. Tukey test was used for all pairwise multiple comparison. *p* value of less than 0.05 was considered statistically significant.

## Data Availability

The datasets used and/or analysed during the current study available from the corresponding author on reasonal request.
